# A case study in the functional consequences of scaling the sizes of realistic cortical models

**DOI:** 10.1371/journal.pcbi.1007198

**Published:** 2019-07-23

**Authors:** Madhura R. Joglekar, Logan Chariker, Robert Shapley, Lai-Sang Young

**Affiliations:** 1 Courant Institute of Mathematical Sciences, New York, New York, United States of America; 2 Center for Neural Science, New York University, New York, New York, United States of America; Peking University, CHINA

## Abstract

Neuroscience models come in a wide range of scales and specificity, from mean-field rate models to large-scale networks of spiking neurons. There are potential trade-offs between simplicity and realism, versatility and computational speed. This paper is about large-scale cortical network models, and the question we address is one of scalability: would scaling down cell density impact a network’s ability to reproduce cortical dynamics and function? We investigated this problem using a previously constructed realistic model of the monkey visual cortex that is true to size. Reducing cell density gradually up to 50-fold, we studied changes in model behavior. Size reduction without parameter adjustment was catastrophic. Surprisingly, relatively minor compensation in synaptic weights guided by a theoretical algorithm restored mean firing rates and basic function such as orientation selectivity to models 10-20 times smaller than the real cortex. Not all was normal in the reduced model cortices: intracellular dynamics acquired a character different from that of real neurons, and while the ability to relay feedforward inputs remained intact, reduced models showed signs of deficiency in functions that required dynamical interaction among cortical neurons. These findings are not confined to models of the visual cortex, and modelers should be aware of potential issues that accompany size reduction. Broader implications of this study include the importance of homeostatic maintenance of firing rates, and the functional consequences of feedforward versus recurrent dynamics, ideas that may shed light on other species and on systems suffering cell loss.

## Author summary

With the vast numbers of neurons in the cerebral cortex, models in neuroscience are, for practical reasons, often orders of magnitude smaller than the actual network. We examine in this article the scalability of cortical networks. We find that function and dynamics in a network depend on network size. For illustration, we use a previously constructed realistic model of monkey visual cortex. Reducing the number of cells in the model, we find that small changes in synaptic weights can help maintain firing rates. However, model characteristics change fundamentally in the reduced models. Neurons have abnormal intracellular dynamics. The model becomes dominated by feedforward inputs and is less capable of functions requiring neuronal interaction. Modelers need to be aware of the potential issues with reduced cortical network models.

## Introduction

One of the greatest challenges of science today is to unlock the mysteries of the brain. The cerebral cortex is a vast network of neurons the dynamical interaction of which shapes our perception of the external world and controls our behavior. Modeling is the key to understanding cortical functions, and to build a model of cortex, one is confronted with the issue of size: the number of neurons in the human cortex is approximately 20 × 10^9^ [1]. In a model with that number of neurons, the costs for simulations can be prohibitive, both in terms of computational resources and time. If one could work with a model that consists of only 1% of the neurons per unit area in real cortex, one would be able to simulate, with similar resources, regions of cortex 100 times larger, or do the simulation 100 times faster.

But can one scale down a cortical network without loss of realism? Are neuronal network models *scalable*, that is, are emergent dynamics retained in network models that are orders of magnitude smaller than the real cortex? To what degree can one scale down a cortical network without impacting its performance or compromising its function? Are some ways better than others to do the downsizing, and are there ways to compensate for inevitable losses? These fundamental questions of theoretical neuroscience motivated this investigation.

There are a number of papers in the literature addressing concerns over the scalability of neuronal networks but none are focused on the important question: how well can one simulate cortical function with models that are reduced in size? Here is a brief sample list of prior work: [2], which investigated and discussed the serious computational issues that arise in realistic modeling of the cerebellar cortex, [3], which studied the scale dependence of mechanisms for initiating oscillatory behavior in certain cortical slabs, and [4], a theoretical study of the dependence of correlations on system size; see also [5]. There also are several theoretical studies in which the authors considered scaling *up*, not down, system size to gain mathematical tractability in the limit as system size tends to infinity; see e.g. [6–8]. That is not the aim of the present paper. Many previous theory papers focused on homogeneous networks of neurons, but realistic models of the cortex are necessarily highly inhomogeneous.

In this paper, we offer insights gleaned from a case study, namely a model of the monkey primary visual cortex (V1), which is very similar to human visual cortex. We chose to use as starting point the model in [9], a model of the input layer to V1 in the magnocellular pathway. The model has been benchmarked using multiple sets of experimental data to reproduce fairly accurately most of V1’s basic spiking and functional properties [9]. It is an ideal starting point because it is realistic, its cell density is the same as that of the real cortex, i.e. it is not scaled down, and it is of a manageable size, representing a handful of the ∼10,000 hypercolumns in monkey visual cortex. Importantly, results of a scalability study will not be confined to the model in [9]. Since all hypercolumns are similar in structure, our findings are immediately applicable to the entire visual cortex, and since cortical regions have a great deal of similarities, we expect our findings to be relevant to other cortical areas.

Starting from the model in [9], we scaled down the network by various factors, tracking its firing rates and other properties. We went beyond previous studies in two different ways: one was to investigate compensation through parameter manipulation; the other was to evaluate and explain the effects of downsizing on functional benchmarks of model performance. We found that cortex operates in a different mode when synaptic current from intracortical interaction is reduced more and more in the down-scaled models, causing some of the V1 properties to degrade. Somewhat surprisingly, we also found that in spite of severely constrained synaptic currents, some important functions of visual cortex can be retained with relatively minor adjustments of synaptic weights.

In addition to answering practical computational questions, scalability studies have the potential to shed light on biology. The moderately scaled down models investigated here can be thought of as models of biological systems damaged due to cell death or dysfunction. Indeed the algorithm we developed for adjusting synaptic weights is analogous to a compensatory process called homeostatic synaptic plasticity known to exist in the real brain [10]. Also, changes in the behavior of scaled down cortices can shed light in unexpected ways on the cortical networks in other species.

## Results

We use as a starting point a previously constructed model of a small piece of the macaque monkey’s primary visual cortex V1 [9]. We will refer to this as the “full-size model”, and study successively more scaled down versions of it.

### The full-size model, size reduction and a first observation

We begin by recalling some basic facts about the model in [9], referring the reader to [9] for more information.

#### Model layout and key parameters

The main component of this model consists of 9 hypercolumns of Layer 4C*α*, the input layer to V1 in the magnocellular pathway. This small piece of cortex represents 0.75 deg × 0.75 deg of the monkey’s visual field at 5 degrees eccentricity. Two other components of the model are LGN (lateral geniculate nucleus), which relays the visual stimuli from the retina to V1, and Layer 6 of V1, which provides feedback to Layer 4C*α* [11].

The architecture and cell density of the network model of Layer 4C*α* follow well documented anatomical facts. We give a sense of the numbers involved, as some of these numbers will be scaled down in the discussion to follow. In the full-size model, each hypercolumn contains ∼ 4000 neurons [12], of which 3000 are excitatory (E) and the rest inhibitory (I). Each E-neuron has ∼200 presynaptic E-neurons and ∼100 presynaptic I-neurons from within this layer. Each I-neuron has 750-800 presynaptic E and ∼100 presynaptic I-neurons. Connectivity is random with the probability of connection decaying with distance [13–15]. The number of LGN cells corresponding to each hypercolumn is ∼10; five are ON (meaning they are excited when the luminance in their receptive field centers changes from dark to light), and five are OFF (meaning they are excited by the change from light to dark). Each neuron in 4C*α* receives input from 4 LGN cells on average; the number can vary from 1 to 6 [16–18]. Each E-neuron in 4C*α* receives input from 40-60 neurons in Layer 6, and each I-neuron receives ∼100 Layer 6 inputs. We have grouped all other modulating forces in a category we refer to as “ambient”.

Each model neuron in Layer 4C*α* receives excitatory input from four sources: (i) ambient, (ii) LGN, (iii) neurons within Layer 4C*α*, and (iv) Layer 6. It also receives inhibitory synaptic input from surrounding I-neurons in Layer 4C*α*. The main parameters of the model are its synaptic coupling weights, representing the total increase in conductance in the postsynaptic neuron caused by each spike. We focus on neurons in Layer 4C*α*, denoting by *S*^*Q*′*Q*^ the coupling from a presynaptic neuron of type *Q* to a postsynaptic neuron of type *Q*′, both within Layer 4. The synaptic coupling from Layer 6 neurons to Layer 4 neurons are denoted $S_{6}^{QE}$. Two other relevant quantities are *S*^*E*,*LGN*^ and *S*^*I*,*LGN*^, the synaptic coupling from LGN to E and I-cells.

Model details, including equations governing the dynamics of membrane potential and conductances, and the model parameters used, are provided in **Methods (Model equations and parameters)**.

The model in [9] is realistic in that it reproduces fairly accurately a wide range of V1 phenomena, all achieved using a single set of parameters. The scalability of several of these properties is studied in this paper. They include firing rates in background and under drive, orientation and spatial frequency selectivity, the production of gamma rhythms, and the presence of simple and complex cells.

*Orientation selectivity* (OS) being one of the most important functions of V1 [19–21], we regard a scaling as acceptable only if OS is preserved. More precisely, the cortical surface of V1 can be thought of as divided into orientation domains arranged around so-called pinwheel centers. Each domain (or patch, as we will sometimes call it) consists of a group of nearby neurons preferring a certain orientation. Upon the presentation of a drifting grating, a domain or patch is optimally driven if it consists of neurons whose preference is aligned with the stripes of the grating, orthogonally driven if its preference is orthogonal to that of the grating. Orientation selectivity means that in response to a grating of high contrast, optimally driven neurons will respond vigorously, while firing rates of neurons in orthogonal patches will not rise significantly above background levels.

#### Size reduction

As mentioned earlier, the model in [9] will be referred to as the “full-size model”, or the “full-size cortex” when there is no ambiguity that the discussion is exclusively about models. The 1/*n*-cortex refers to a model in which Layer 4C*α* and Layer 6 each has 1/*n* the number of neurons in the full-size cortex. More precisely, neurons in each layer of the full-size cortex are assumed to be located in a 2D rectangular lattice, and the 1/4-cortex, for example, is obtained from the full-size one by choosing a sublattice having half as many neurons in each direction. The probabilities of connectivity as function of distance are retained, so each neuron in a 1/*n*-cortex will have 1/*n* the number of presynaptic neurons.

A choice had to be made with regard to the external sources of input, namely LGN and ambient. For the 1/*n*-cortex, one possibility was to reduce the *total* amount of LGN and ambient drive to the model cortex by a factor of *n* while retaining the same level of LGN and ambient drive to each neuron; the other was to reduce the amount of drive for each neuron by a factor of *n*, i.e., by a factor of *n*^2^ in total input. We chose the first both because it seemed more natural and because there are difficulties associated with reducing the amount of LGN input to individual neurons: First, each neuron in Layer 4C*α* receives on average only 4 LGN inputs, the configuration of which is crucial for the neuron’s orientation selectivity; and second, we found that even if we were to scale down LGN and ambient drive some other way, e.g., by their currents, a 30% reduction would cause spontaneous activity in cortex to cease, and that is pathological.

In the rest of this paper, then, a 1/*n*-cortex refers to a model in which cell densities in Layer 4C*α* and Layer 6 of V1 are reduced by a factor of *n*, as are the total LGN and ambient inputs into 4C*α*, while these inputs to individual neurons are unchanged from the full-size cortex.

#### Firing rates of reduced cortices

**Fig 1:**
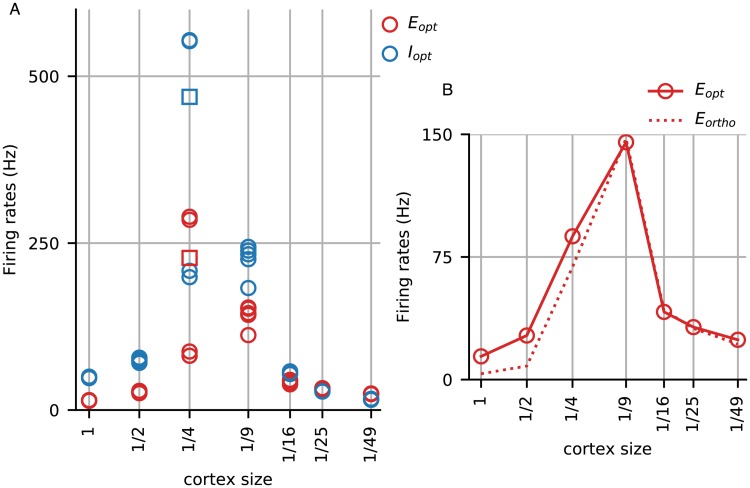
Firing rates without compensation in model cortices of various sizes. **A**. Mean firing rates of Excitatory (red) and Inhibitory (blue) neurons in the optimal patch in response to a vertical grating at high contrast. Firing rates for the full-size cortex are shown, as are those for the 1/2, 1/4, ⋯, 1/49-cortices using identical synaptic weights as the full-size cortex. Five trials (different networks with different initial conditions) were run for each cortex size. Each trial was run for 7 sec, and the open circles denote the E and I-firing rates averaged over the last 5 sec. All trials had converged long before the end of the 7 sec except for one of the trials for the 1/4-cortex (red and blue squares). Results across trials for each cortex size were consistent except in the case of the 1/4-cortex, which had two limiting values; the 1/9-cortex also showed greater variations than others. **B**. The two graphs show optimal (solid) and orthogonal (dashed) firing rates of E neurons in the models. Optimal and orthogonal firing rates were far apart in the full-size and 1/2-cortices, suggesting orientation selectivity. OS was lost for the 1/4- and smaller cortices. All other trials showed similar results with regard to OS.

We start by examining the firing rates of reduced models using the synaptic coupling parameters of the full-size model. That firing rates are important needs no justification as they carry the main information passed from one region of cortex to another. One might expect that a simple “scaling down” with fewer presynaptic neurons, hence less synaptic current, would cause firing rates to go down with cortex size. However, as Fig 1 shows, this is far from the case.

Focusing first on firing rates in the optimally driven patch (Fig 1A), we observe that Excitatory-cell firing rates in all of the reduced cortices are higher than in the full-size cortex, as much as 10 times higher in some of the intermediate size cortices: for example, mean firing rate rises from around 15 spikes per sec in the full-size cortex to 150-300 spikes/s in the 1/4- and 150-200 spikes/s in the 1/9-cortices before dropping off. We note also that the ratio of Inhibitory to Excitatory-cell firing rates, which was 3-4 in the full-size cortex, decreases monotonically with cortex size until it drops below 1 in the 1/49-cortex. Fig 1B shows that orientation selectivity was lost as cortex was downsized: from the 1/4-size cortex on, firing rates in the orthogonal patch are comparable to those in the optimal patch, showing clearly a lack of orientation selectivity.

Explaining the results in Fig 1 analytically is beyond the capability of existing techniques. Simple mean field linear equations clearly do not apply. We cannot avail ourselves of standard theoretical methods such as continuum limit arguments [21–23] as our models are finite-size to begin with and getting smaller, nor can we appeal to sparse-coupling ideas [6], as our connection probabilities are quite far from zero and do not tend to zero. Most important is that all of the main theory papers that we know of are about homogeneously connected populations with homogeneous drives. In our model as in real cortex, neurons have spatial locations, connectivity varies with distance, and visual stimuli affect neurons in the model (as in the real visual cortex) in highly inhomogeneous ways. These features lead to different firing rates across the cortical surface and considerably more complex dynamics than in homogeneous population models.

What makes the analysis challenging are the recurrent interactions between E and I neurons. For example, when excitation in a local population is increased, the change will affect both E and I-neurons, causing, as a first reaction, both populations to have higher firing rates. But then these two populations interact: the E-neurons will excite further both themselves and the I-population while the I-neurons try to suppress the E-neurons. The net result of this interaction is hard to analyze in this model that also simulates accurately the spatial inhomogeneity of the cortex: to complicate matters, there is the influence of nearby neurons, which the stimulus may have affected in different ways. Changes in model conditions cause the local E- and I-populations to “renegotiate” a new balance point, the value of which is not easy to predict.

While it is beyond the scope of this paper to provide a rigorous analysis of why the reduced models become non-functional, we have observed the following in many simulations of the model: If suppression in the domains representing non-preferred orientations is too weak relative to the tendency for excitation to self-promote, then the region of excitation can spread beyond the intended orientation domain. It will then invade the entire model cortex causing firing rates to increase uniformly everywhere. This is how orientation selectivity is lost. A phenomenon characteristic of recurrent models is that recurrent excitation can be set off by a slight deficit in inhibition in the E/I balance, and once it takes off, is hard to control. Generally, models with high gain such as the one being investigated here are inherently somewhat unstable.

The simulations in Fig 1 were performed with multiple trials and careful studies of convergence properties. These results show that if one takes a full-size cortex such as the one in [9] and scales it down without adjustments in synaptic weights to compensate for current changes, the behavior of the model cortex can be altered dramatically. Indeed Fig 1 shows that from the 1/4-size cortex on, the reduced models were hardly recognizable as copies of the full-size model cortex in terms of firing rates and orientation selectivity, two of V1’s most important functions. In this sense, cortical networks are not scalable.

The scaled down cortices are not intended as realistic depictions of a pathological cortex suffering the effects of cell loss due to death or dysfunction. Nevertheless, the dramatic changes in model features between the 1/2- and 1/4-cortices suggest that cell loss on a similar scale in the real cortex—without compensatory measures—can have very severe consequences. We will, in the next section, allow parameter changes to compensate for the effects of size reduction.

### Restoring firing rates through synaptic compensation

Now that we know that in the cortical model without compensation the firing rates of the scaled-down models deviate from those in the full model, we seek to restore functionality by keeping local population firing rates as close to those of the full-size model as possible, and to do that by manipulating the synaptic weights in the scaled-down models. *A priori* there are many ways to do this. For example, compensating for the loss of presynaptic neurons by increasing synaptic weights by the same factor might seem at first sight to be a natural solution, but that produces pathological behaviors, e.g., with a 10-fold reduction in model size, hence a 10-fold increase in synaptic weights, 2-3 spikes from its set of presynaptic E-cells in quick succession will cause a postsynaptic neuron to spike. To have the potential to shed light on biology, we must modify synaptic weights as the biological cortex might do. Thus we seek to locate regimes for reduced cortices that produce local population firing rates similar to those in the full cortex, and to do so in a way that *minimizes changes in synaptic weights*. We targeted firing rates because keeping firing rates in a narrow range seems to be the goal of homeostatic synaptic plasticity [10].

We focus on the following 6 quantities

$$\begin{array}{c} (E_{\text{opt}},\;I_{\text{opt}};\;\;E_{\text{ortho}},\;I_{\text{ortho}};\;\;E_{\text{bg}},\;I_{\text{bg}}),\; \end{array}$$ (1)

where *E*_opt_, *I*_opt_ are mean E- and I-firing rates (averaged over all E, I neurons respectively) in an optimally driven or vertical-preferring region of the model when presented with a high contrast drifting grating of vertical orientation; *E*_ortho_, *I*_ortho_ are mean firing rates in the corresponding orthogonal (ortho) or horizontal-preferring region, and *E*_bg_, *I*_bg_ are background firing rates. For the full size model, these numbers, in spikes/sec, are

$$\begin{array}{c} (14.71,\;51.15;\;\;3.72,\;21.48;\;\;2.93,\;11.35).\;\; \end{array}$$

To determine the viability of size reduction, our first criteria were that the firing rates of the reduced models fall in the following ranges:

1. *E*_opt_ ∈ (13, 16),
2. *E*_bg_ ∈ (1.5, 4), and
3. *I*_opt_/*E*_opt_, *I*_bg_/*E*_bg_ ∈ (3, 5).

We seek to maximize *E*_opt_/*E*_ortho_ without setting a bound, regarding a ratio of 3 as strong OS and < 2 as compromised.

#### Algorithm for parameter adjustment in reduced cortices

We propose in this paper an algorithm for adjusting the synaptic weights in reduced models systematically to achieve desired firing rates. Two quantities central to this algorithm are diff_*E*_ and diff_*I*_, defined as follows:

$$\begin{array}{c} {\text{diff}}_{E}\;:=\;\text{(total)}\;\text{E-current}\;\text{into}\;\text{E-cells}\;-\;\text{I-current}\;\text{into}\;\text{E-cells}\;. \end{array}$$

The equation is in normalized units (see **Methods, Model equations and parameters**), and it will be applied to E-cells in a local population under a particular set of circumstance, e.g., the model is driven by a high-contrast vertical grating, and the patch in question is the optimally driven patch in Layer 4C*α*. Current refers to the mean current entering an E-neuron in the patch, averaged over all neurons in the patch and over time (over the last 5 sec of a 7 sec run); total E-current refers to the sum of the currents from Layer 4C*α*, Layer 6, LGN and ambient; I-current comes only from Layer 4C*α*. The quantity diff_*I*_ is defined analogously:

$$\begin{array}{c} {\text{diff}}_{I}\;:=\;\text{(total)}\;\text{E-current}\;\text{into}\;\text{I-cells}\;-\;\text{I-current}\;\text{into}\;\text{I-cells}\;. \end{array}$$

The reason these are important quantities is that neglecting the leak term, diff_*E*_ (respectively diff_*I*_) is the right hand side of the the equation governing the time evolution of membrane potential dynamics (see **Methods, Model equations and parameters**) for an E (respectively I)-neuron. That is to say, by integrating this quantity over a period of time, we will obtain the number of times the neuron crosses its spiking threshold during this period, hence its firing rate. Thus for the smaller cortices to have the same firing rates as the full-size cortex, it would be logical, as a first approximation, to aim for the same value of diff_*E*_ and diff_*I*_ observed in the full cortex. On the other hand, since the total E and I-currents produced by the cortex are proportional to system size, we can try to scale the synaptic weights accordingly to produce the same current differences (current *differences*, not currents). This, together with a small trick to improve orientation selectivity is, in a nutshell, the algorithm we propose.

Because of our interest in keeping the synaptic weights close to those in the full-size cortex, instead of looking directly at synaptic couplings *S*^*Q*′*Q*^, *Q*, *Q*′ = *E*, *I*, for the reduced cortices, we will focus on the multiplicative factors

$$\begin{array}{c} m_{Q^{\prime }Q}\left(n\right)=\frac{S^{Q^{\prime }Q}\;\text{for}\;\frac{1}{n}\text{-cortex}}{S^{Q^{\prime }Q}\;\text{for}\;\text{full}\;\text{cortex}}\;. \end{array}$$

To reduce the number of parameters to be varied, we assume in the reduction process that $S_{6}^{Q^{\prime }Q}$ be indexed to *S*^*Q*′*Q*^ the way it is in the full cortex.

Details of the algorithm are given in **Methods (Algorithm for synaptic weight compensation)**.

We stress that there is nothing *a priori* to guarantee that the algorithm proposed will work, or that firing rates in reduced cortices can be put in the desired ranges at all. The problem consists of steering 6 numbers, namely the 6 quantities listed in (1) above, into target regions by manipulating the 5 synaptic weights, *S*^*EE*^, *S*^*EI*^, *S*^*IE*^, *S*^*II*^ and *S*^*I*,*LGN*^. Recall that there is no explicit formula linking the target quantities to the parameters to be varied. Firing rates are determined by a neuron’s intrinsic properties together with the synaptic inputs it receives, and the latter is in turn determined by the emergent dynamical interaction among neurons in the network. Also, the 6 quantities above are not independent; they are the firing rates, under two stimulus conditions, of 4 groups of neurons in a highly interconnected network, and are related in very complicated ways through their interaction.

As we show next, in spite of the theoretical difficulties, the compensation algorithm worked surprisingly well in bringing the reduced cortical models into acceptable ranges of firing rate and OS.

#### Application of algorithm and preliminary results

Applying the simple algorithm above to 1/*n*-cortices for *n* = 2, 4, 9, 16, 25, 49, we were able to locate in each case regimes with firing rates in the acceptable ranges. The results are summarized in Table 1.

**Table 1:** Synaptic weights and restored firing rates of reduced cortices. This table summarizes the results of applying the algorithm in **Results (Restoring firing rates through synaptic compensation)** to identify example model cortices of successively smaller sizes. The seven example cortices the data of which are shown below will be used in studies in the rest of the paper. All have firing rates within prescribed ranges. The algorithm favored keeping the multiplicative factors *m*_*Q*′*Q*_ close to 1 and maximizing OS, but no optimization procedure was expressly carried out for those purposes.

$\frac{1}{n}$-cortex	*m*_*EE*_	*m*_*EI*_	*m*_*IE*_	*m*_*II*_	*m*_*I*,*LGN*_	*E*_opt_	*I*_opt_	*E*_ortho_	*E*_bg_	*I*_bg_	$\text{OS}=\frac{E_{opt}}{E_{ortho}}$
n = 1	1	1	1	1	1	14.71	51.15	3.72	2.93	11.35	3.95
n = 2	1.03	1	1.24	1	1	14.35	53.85	4.51	3.14	11.89	3.18
n = 4	1.09	1	1.68	0.8	1.1	13.85	68.57	4.46	2.72	11.23	3.10
n = 9	1	1.44	1.71	0.5	1.3	14.01	49.87	4.57	2.03	8.47	3.07
n = 16	1	2.36	1.18	0.7	1.4	13.23	40.4	4.41	1.69	8.24	3.00
n = 25	1	3.11	3.4	0.6	1.3	13.17	47.05	5.86	1.79	8.17	2.25
n = 49	1	4.17	7.02	0.8	1.4	14.02	52.6	6.58	2.3	9.79	2.13

Observe that OS, quantified by *E*_opt_/*E*_ortho_, deteriorated steadily from ∼ 4 in the full-size cortex to ∼ 2 in the 1/49-cortex, the numbers remaining ≥ 3, which we consider to be satisfactory, through *n* = 16.

Up through *n* = 16, the modifications in synaptic weight needed were remarkably small: for *n* ≤ 9, all multiplicative factors m_*,*_ were ∈ (0.5, 1.7), and at *n* = 16, the largest was *m*_*EI*_ = 2.36. These values are not far from the degree of adaptation seen in real neurons [24]. That such a small modification of synaptic properties was sufficient for restoring firing rates and orientation selectivity in systems an order of magnitude smaller is a demonstration of the power of homeostasis: when performed judiciously, a few small changes in the intrinsic properties of the neurons can go a long way.

We stopped at the 1/49-cortex, which has roughly 2% the number of neurons as the full cortex and only two presynaptic I-neurons for each neuron. For this cortex, *m*_*IE*_ became too large for the system to be regarded as being related to the full-size cortex.

*The example cortices shown in* Table 1 *will be used in our scalability study in the rest of this paper*.

**Fig 2:**
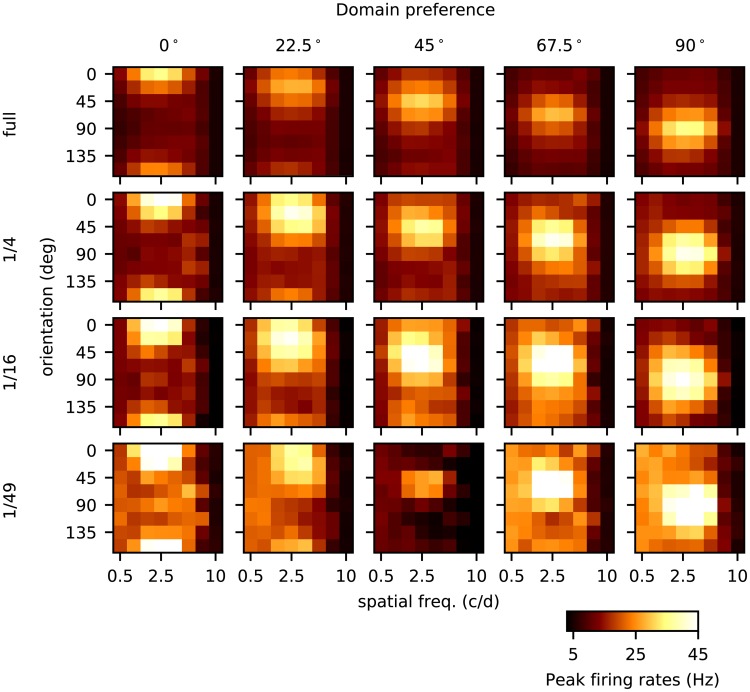
Orientation and spatial frequency tuning for four model cortices. This figure shows 20 panels each one of which gives the responses of a group of neurons to 64 different gratings. Each row represents responses of a different size cortex: full-size, 1/4-, 1/16-, and 1/49-cortices from top to bottom. In each model cortex, we divided the region around the pinwheel center into 8 wedges evenly spaced, and the 5 columns show responses from E-neurons in wedges that, if all went according to plan, would have orientation preferences 0°, 22.5°, 45°, 67.5° and 90° respectively. Within each panel are responses to 64 gratings drifting across the visual fields of the model cortices at 4 Hz. In the *x*-axis are the spatial frequencies of the grating ranging from 0.5 to 10 c/d, and in the *y*-axis are orientations of the gratings from 0° to 157.5° evenly spaced. The color of each little square represents the peak firing rate averaged over the E-neurons in that wedge; see color bar at the bottom. For the full cortex, one sees that the strongest responses, from left to right for rows 1–5 of each panel, coincide with the intended preference of the wedge. The same is true for the other three cortices. The preferred SF for this layer of V1 is between 2-3 c/d [20], which corresponds to the third to fifth columns from the left within each panel. Both orientation and SF preferences are correctly reflected in all 20 panels, indicating that all four cortices have at least moderate to strong orientation and SF selectivity. But there are also differences among the cortices of different sizes: One is that *peak* firing rates are, on average, significantly higher in the smaller cortices (even though by design all have similar *mean* firing rates). The second is that the 1/49-cortex shows greater inconsistency in its response across the 5 wedges.

As an initial assessment of the performance of the reduced cortices with compensation via adjustment of synaptic weights, we examined two basic properties of V1 neurons in these cortices: orientation and spatial frequency (SF) tuning. As in [9], we presented to each of the model cortices 64 drifting gratings corresponding to 8 different orientations and spatial frequencies, and recorded peak firing rates from E-neurons in 5 patches consisting of neurons with different intended orientation preferences. The results are shown in Fig 2.

Details of the plots are explained in the legend of Fig 2. We summarize the main points of note: One is that neurons in cortices of all sizes clearly have OS and SF tuning. For OS the results in Fig 2 go beyond those in Table 1 as we will explain in the next paragraph. Second is that though all showed OS and SF tuning, something was clearly different as cortex size was decreased, that is, the four rows in Fig 2 are far from identical; and third, there was obvious degradation as cortex size was shrunk; behavior was more erratic in the 1/49 cortex.

In the model in [9], each hypercolumn was divided into 6 wedges arranged around a pinwheel center. Following [19], neurons in each of the wedges were connected to a different set of LGN templates of ON and OFF cells aligned in a certain direction. These templates favored 0°, 30°, 60°, 90°, 120° and 150°. In Fig 2, we have deliberately used 8 gratings that are evenly spaced to cover the same angular range, hence incommensurate with the 6 templates. This means in particular that for the three wedges preferring 22.5°, 45° and 67.5° (the middle three columns), in order for neurons to respond most vigorously to the grating intended to evoke the strongest spiking, they had to “average” the feedforward inputs received through dynamical interaction among cortical neurons. Since the vertical spread of responsivity in the panels in Fig 2 for these wedges are not larger than those for the wedges preferring 0° and 90°, we conclude that this averaging was done successfully to some degree in spite of the shrunken cortex size.

### Similar yet different

The aim of Part III is to take a more in-depth look at the internal workings of the different model cortices, to discover what changed as cortex size was scaled down. Recall that our compensatory algorithm was designed to bring the mean population firing rates in optimally and orthogonally driven regions to conform with those of the full-size cortex. Here we report on the accompanying changes in dynamics without further tuning of parameters.

#### From cortical processing to a simple feedforward relay

Even though they produced mean firing rates similar to the full cortex, the smaller cortices after compensation had a distinctly different character. Many of the differences are rooted in the fact that they were increasingly dominated by feedforward LGN inputs, so we begin by documenting this fact.

**Fig 3:**
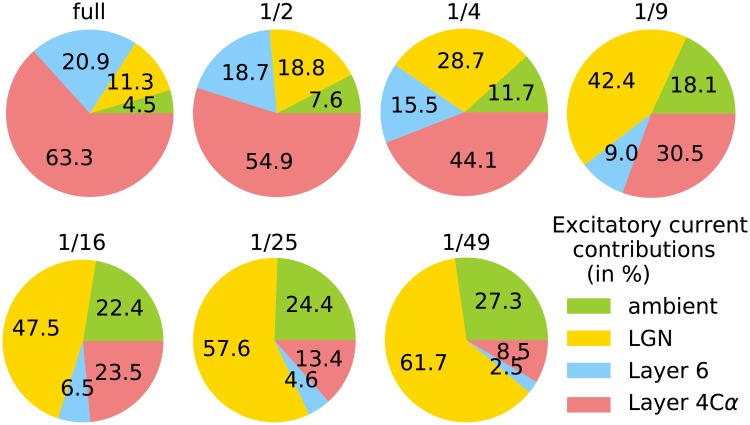
Excitatory current decomposition across various cortices. The piecharts show the contributions from various sources of excitatory current into E-cells in optimally driven regions, during the presentation of a drifting vertical grating at high contrast. The size of the cortex is indicated above each piechart. Current sources: LGN (yellow), ambient (green), local cortical circuit Layer 4C*α* (red), cortical feedback from Layer 6 (blue). The numbers were computed from simulations of the reduced cortices. Simulations are run for 5 sec after ignoring the first 2 sec.

A traditional view from the time of Hubel and Wiesel had been that input layers of the sensory cortices were largely driven by feedforward inputs, with cortex exerting mostly modulatory influences [19, 25]. Over the years, it was realized that the fraction of feedforward input was not as large as once thought [26]. Following data from [16–18], it was found in the model of [9], i.e., in the full size model in this paper, that the bulk of the input current to cells in Layer 4C*α* comes from cortex, most of it from the local circuit and a smaller fraction from Layer 6, with only 11% from LGN and a few percent from ambient, modulatory sources. This is depicted in the upper-left piechart in Fig 3. For a Layer 4C*α* neuron in the 1/*n*-cortex, the number of presynaptic neurons was scaled down by a factor of *n*, while its LGN and ambient input was unchanged. It is therefore not surprising that the fraction of current from cortex decreased steadily with cortex size, from 84% in the full cortex to only 11% in the 1/49 cortex, while the fraction of its drive from LGN rose from 11% to 62%, making the system increasingly feedforward driven.

#### Changes in intracellular activity

**Fig 4:**
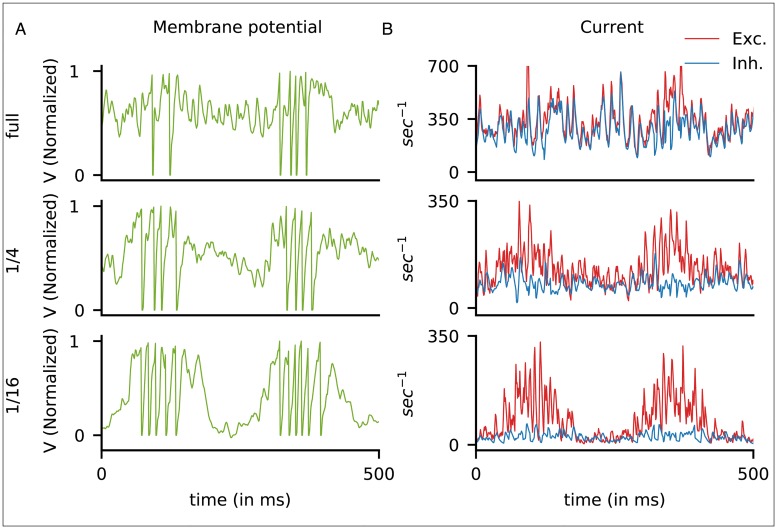
Membrane potentials and current traces of model neurons. Cortex sizes were full (top row), 1/4-(middle) and 1/16-(bottom). A. Membrane potentials and B. current traces of three representative model E-neurons, all simple cells as evidenced by their patterns of spiking in response to a 4 Hz grating at high contrast. The membrane potentials are normalized with spike threshold at 1. Excitatory current (in normalized units) is in red, and Inhibitory current in blue. Observe that in the full cortex, E and I-currents covaried and nearly coincided, except when spikes were fired, while in the 1/16-cortex, E and I-currents were decoupled, and E-current peaks coincided with the peaks of LGN cycles.

The change in the character of cortical processing in the scaled-down models is clear when one compares the intracellular electrical activity of cells in the full and scaled-down models. Close-up looks at the membrane potentials and synaptic currents of representative neurons from the full, 1/4, and 1/16-cortices are shown in Fig 4. In addition to differences in magnitudes of the currents (which one expects as cortex size reduces), these plots show strong qualitative differences: In the full cortex, E- and I-currents were comparable in magnitude, and they covaried, i.e., they went up and down together. Momentary excesses of E-currents led to spikes. This picture is consistent with data from [27]. In the 1/4-cortex, the discrepancy between the E- and I-currents was larger, especially during LGN peak periods when most spikes were fired. In the 1/16-cortex, I-current hit the floor, and E-current from cortex alone was insufficient to cause spikes. This is evident from the fact that barrages of spikes were fired 250 ms apart, consistent with the hypothesis that spike firing occurred exclusively during the peak cycle for LGN in response to a grating drifting at 4 Hz. During the peak of an LGN cycle, LGN supplied enough drive to initiate the spiking, and once spike firing started, it was magnified by recurrent excitation.

Fig 4 thus shows clearly that the fraction of feedforward input has a strong effect on the patterns of current dynamics. Even when compensated to produce the correct firing rates, the severe reduction in current in the smaller cortices disrupted nontrivially the E/I balance in the full cortex. As noted in [28], the model [9] is in a tightly balanced state when driven by visual stimuli. This fact is evident in Fig 4B, top panel, where E and I-currents track each other precisely. In the scaled down cortices, mean I-current is a small fraction of mean E-current during peak LGN cycles, as shown in Fig 4B, middle and bottom panels. Not only are E and I currents not tracking one another, here the model is no longer in a balanced state in the sense of [7], though it continues to produce reasonable firing rates and to have orientation selectivity.

#### Changing population dynamics

**Fig 5:**
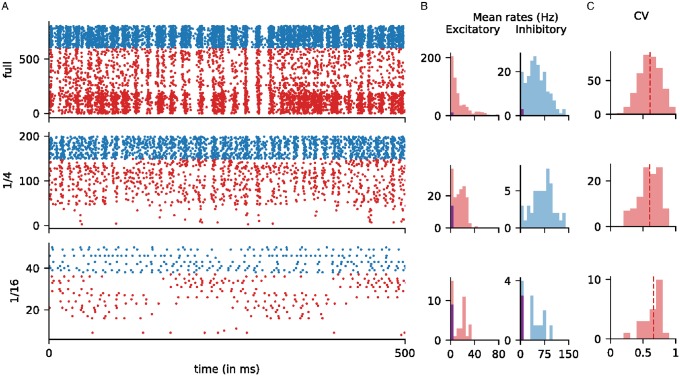
Population dynamics in model cortices of various sizes. Statistics for three model cortices are shown: the full-size cortex (top row), 1/4-cortex (middle) and 1/16-cortex (bottom). **A**. Rasters of neurons in an optimally driven patch when driven by a high-contrast grating. The *x*-axis is time, and the *y*-axis is neuron number, and dots indicate when spikes occurred. Excitatory neurons are in red; Inhibitory ones are in blue. The E-neurons are ordered by the number of LGN inputs, from 1 LGN (bottom) to 6 LGN (top). **B**. Distributions of mean firing rates of the E and I neurons in the same patch. The purple bars on the left of each histogram indicate silent neurons, i.e., neurons that did not fire. For the smaller cortices, the E-neuron distributions show bimodality, as neurons with fewer LGN inputs became increasingly silent. **C**. Distributions of CV of the E neurons in the same optimally driven patch. Only those neurons with firing rates above 5 Hz are shown.

We now examine how the reduction in cortex size affected the dynamical interaction among neurons. Rasters of optimally driven patches are shown in Fig 5A for the full, 1/4 and 1/16 cortices. These three example cortices give a good idea of the trends in changing population dynamics as *n* increases.

*Weakening and disappearance of gamma-band rhythms*. Partial synchronization of spike firing in the gamma band is clearly observed in the full-size cortex model as in the real brain [28–30]. This synchronized activity is weakened but still present in the 1/4 cortex, and is absent in the 1/16 and smaller cortices (Fig 5A).

We explain why models that are too small in size cannot support realistic gamma-band rhythms: Recall that gamma-band rhythms are strongly mediated by recurrent excitation, which causes a wave of spiking by both E and I-cells followed by pushback by the latter leading to a momentary lull in the spiking [9, 28, 31]. In the full-size model, the synchronization is very partial: typically no more than 10-15% of the local Excitatory-cell population, or 20-30 E-neurons, together with a commensurate number of I-neurons, participate in each firing event blue [9, 28]. As it would be difficult for very few neurons (e.g. 2-3) to precipitate recognizable spiking events, these events would either have to disappear in smaller cortices or they would have to involve more substantial fractions of the local population, producing more synchronized spiking. Increased synchrony was not seen in our reduced model cortices.

It has been suggested that gamma-band rhythms may play a role in communication among cortical regions [32]. If that is the case, then unlike full-size cortices, smaller networks will lack the ability to communicate between regions.

*Shifting firing rate distributions and loss of complex cells*. In the rasters in Fig 5A, we ordered the excitatory cells by the number of LGN inputs. The bottom 30% of the E-cells received 2 or fewer LGN inputs and were given higher cortico-cortical connectivity to compensate. In the full cortex, i.e., in [9], the bottom 30% corresponded roughly to complex cells [33, 34], or cells with Modulation Ratio < 1, and their mean firing rates were roughly three times those of simple cells (following data). See the top raster in Fig 5A, where the spike firing patterns of the bottom 30% of cells have a distinct look. In the two smaller cortices, even with the same elevated probabilities of connection, firing rates of cells with low LGN input, the bottom 30%, were reduced drastically due to insufficient drive from cortex. In the 1/16-cortex, most cells with 1 or 2 LGN inputs became silent, and nearly all cells were simple.

One of the functions of complex cells may be to smooth out the response of simple cells, which tend to follow closely the rises and falls of light intensity in the gratings. Our study shows that this feature will be lost in smaller cortices.

The compensation algorithm we used was designed to produce regimes the mean firing rates of which satisfy certain conditions; e.g., we required that *E*_opt_, the mean firing rate in response to a grating at high contrast, lie between 13–16 spikes/sec when averaged over neurons in an optimally driven patch. No attempt was made to control firing rate distributions within the patches. As cortex size decreased and complex cells went silent, to maintain population means, excitatory cells with larger number of LGN inputs had to fire more. This expectation is consistent with Fig 5B, which shows that excitatory firing-rate distributions became increasingly bimodal, separating into active and inactive groups.

The cell depicted in Fig 4 for the 1/16-cortex clearly belonged to the high firing group, as can be seen from the large burst that occurred when the LGN cycle was favorable. Such characteristic bursts that follow LGN closely can be seen in the corresponding raster in Fig 5A. The timing of these bursts depends on when the bulk of the LGN input is received, which is different for different cells. The group structure observed in the firing patterns for the 1/16 cortex depends on ordering of the cells and is accidental.

*Circular variance*. Fig 5C shows the distributions of circular variance (CV), a widely used measure of orientation selectivity [35]. CV varies from 0 to 1, with 0 indicating perfectly selective and 1 indicating nonselective neurons. In the full-size model, mean CV was between 0.6 and 0.7 and the distribution had a good spread [9], indicating varied degrees of tuning and diversity; this is consistent with data [35]. These statistics did not change substantially with cortex size: the amount of tuning for all 3 cortices shown are similar, and neurons continued to show diversity even as cortex reduced in size.

*Fine-scale orientation preference maps*. LGN cells being very sparse, they represent only a few discrete angles. Following [9], these angles in our models are 0°, 30°, 60°, 90°, 120° and 150°. That means all simple cells in layer 4*Cα* receive LGN inputs favoring one of these 6 angles. We now investigate the preferences of cortical neurons, to see how they respond to angles in between.

**Fig 6:**
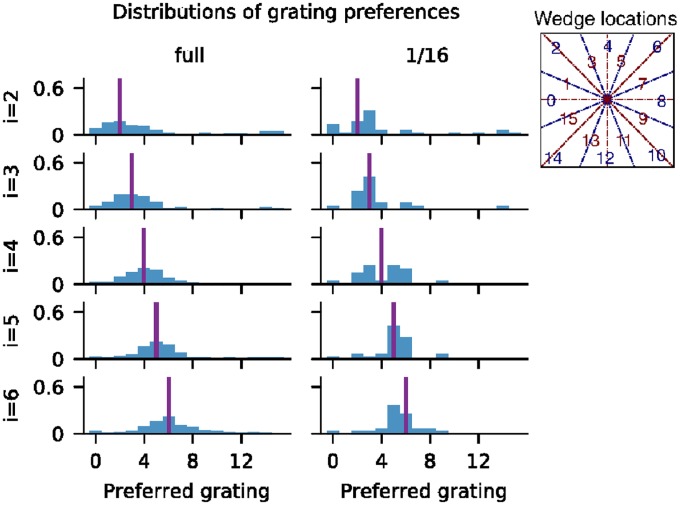
Fine-scale orientation preferences. Normalized histograms showing the preferences of neurons in wedges 2–6, for the full cortex and the 1/16-cortex. The *i*th row shows data for wedge *i*; *x*-axis shows grating numbers. Purple line represents the intended orientation preference at the center of the wedge. Inset shows wedge locations in the central hypercolumn of the 9-hypercolumn model.

We divided the central hypercolumn of the model cortices into 16 overlapping wedges, organized around the pinwheel. These wedges are labeled 0, 1, ⋯, 15, and their centers are as shown in Fig 6 (inset). We considered also 16 gratings at full contrast, labeled 0, 1, ⋯, 15, with angles evenly spaced between 0° and 180°. These gratings are aligned in such a way that if there was a mathematically precise relationship between angles around the pinwheel and orientation preferences of neurons at those locations, then neurons at the center of wedge *i* would prefer grating *i*. Because the wedges overlapped, neurons in wedge *i* would in fact prefer grating *i* with probability $\frac{1}{2}$, and gratings *i* − 1 and *i* + 1 each with probability $\frac{1}{4}$. (We used overlapping wedges to ensure a large enough sample, as neuronal response was very diverse at least in the full-size cortex [9]).

The PDF’s of preferences for neurons in wedges 2–6 for the full and 1/16-cortices are shown in Fig 6, the purple line in each panel indicating the angle of the grating associated to the center of that wedge. Cursory inspection tells us that none of the PDF’s shows a perfect match but all are reasonable approximations. In particular, the distribution is smoother but wider in the full cortex, some having a shape resembling a Gaussian.

Let us now examine the situation more closely. Grating 4, which corresponds to 45°, is particularly interesting, because it is farthest away from the angles represented by LGN. In the full cortex, many neurons preferred this grating, while few did from the 1/16-cortex, not even neurons in wedge 4. Observe also that while the distributions were fairly symmetric about the intended orientation for the full-size cortex, the preferences of neurons in the 1/16-cortex were biased: Neurons in wedges 2 and 3 had very similar preferences, both locked onto the 22.5° and 34° gratings with some preference for the latter, suggesting an affinity for 30°, one of the angles represented by LGN. Similarly, wedges 5 and 6 showed a preference for 60°, another LGN angle, and when the theoretical preference fell squarely between these two angles, as in wedge 4, then the preference was bimodal: half of the neurons behaved like those in wedges 2 and 3, and half like those in wedges 5 and 6.

The fine-scale orientation preference shown by neurons in the full-size model illustrates its ability to transform the input signal, a capability lacking or compromised in the 1/16-cortex, leading to a less smooth orientation map. No doubt this has to do with the dominance of LGN as a source of excitatory input for the 1/16-cortex. It was also likely exacerbated by the loss of complex cells, which, our analysis showed, contributed nontrivially to filling in the gaps.

## Discussion

In engineering, business, or other projects of human design, scalability studies are standard: one first establishes feasibility in a small model before scaling it up. In science, we have to face the complexity of nature as it is. To gain insight into nature one often has to work with models that are orders of magnitude smaller and simpler, and an important question is to what degree these reduced models can reflect nature’s design faithfully. Questions of scalability are therefore equally pertinent in the modeling of natural phenomena, but here one asks if nature’s design can be scaled *down*.

The aim of the present article is to investigate the feasibility of scaled down models in neuroscience, and to call attention to some issues associated with size reduction. This paper is a case study of scalability, that of a realistic model of the monkey visual cortex [9]. The model in [9] is realistic in the sense that it is true to scale and has been benchmarked to reproduce many of V1’s basic properties. We have used it as our starting point and studied whether or not it could be downsized without unacceptable loss of function. Although this is just an example, the model architecturally has much in common with many other neuronal networks in the cerebral cortex, and the main messages of this paper, which we now discuss, are relevant to a broad range of biological networks.

### Scalability of cortical networks: Lessons from a case study

Scaling down the model cortex in [9] without compensation caused a major disruption of function (Fig 1). As to how to compensate, it seemed paradoxical *a priori* how one could coax a 1/*n*-model, e.g. *n* = 16, to produce firing rates similar to those of the full-size cortex when each neuron received input from only a few percent of its original set of presynaptic neurons. Would it mean synaptic weights had to be increased *n*-fold? The answer was certainly not; we were able to manage with much smaller changes (Table I). This is because spike firing has little to do with absolute values of Excitatory and Inhibitory currents; it has to do with the *difference* between E and I; see [7] for related ideas. Controlling current differentials was the guiding principle of the compensatory algorithm we used: by forcing the smaller cortices to preserve current differences (diff_*E*_ and diff_*I*_) similar to those in the larger cortices, we put them in a regime not far from the desired ones, within range for us to steer the dynamics systematically towards the target regimes. The procedure we used enabled us to downsize the model cortex in [9] up to a factor of ∼ 50 preserving mean firing rates in local populations, orientation and spatial frequency selectivity.

Our first message can therefore be summarized as follows: *neuronal networks can tolerate a fairly drastic reduction in current and continue to produce reasonable firing rates and relay signals provided one allows moderate changes in synaptic weights to compensate*. In this sense, cortical network models are quite scalable.

But then there is the following caveat: scaled-down networks are not just smaller copies of the original network; they can have substantially different internal dynamics. In our case study, even as the reduced cortices were able to perform their primary functions of orientation and spatial frequency selection (Fig 2), the electrical properties that govern spike firing of neurons were changed (Fig 4), and the composition of the current that drove the entire system shifted toward domination by a feedforward drive and away from cortico-cortical interaction (Fig 3). Since a system cannot function with insufficient drive, one has to expect the fraction of feedforward input to be larger and larger as we scale down a model. These changes together with diminished intracortical interaction resulted in the weakening of gamma rhythms and the loss of complex cells (Fig 5), both of which involved dynamical interaction among neurons within cortex. Neurons in reduced cortices also lacked the fine-scale orientation preference seen in the full-size cortex (Fig 6).

This leads to our second message, which is that *even when a reduced network is able to relay its input signals to the next region, there is the possibility that its ability to transform the signal suitably may be compromised*. In our case study there were clear indications that diminished lateral dynamical interaction in the reduced cortices had consequences. We did not go beyond the properties modeled in [9] but V1 has many other functions, e.g. contrast response, directional selectivity, surround modulation, binocular vision, and even initial stages of motion detection, to mention just a few. The issues discussed above are by no means confined to the visual cortex. Findings from the present study suggest that caution should be exercised with regard to functions that required lateral dynamical interaction when using network models that are reduced in size.

### Broader implications

#### A catalogue of recurrent vs feedforward networks

Although our motivation for exploring scaling down the size of cortical networks was to understand the functional, dynamic consequences of scaling the network size, in fact what we did also was to develop an understanding of the dependence of network behavior on the feedforward-to-recurrent ratio of the local population. We created a catalogue of cortical networks that varied systematically in this ratio. This catalogue is a useful analytical tool for evaluating and comparing cortical network models and, through the analysis of the models in the catalogue, also evaluating and comparing cortices in different species—or possibly the same cortex in different developmental or functional states.

For example, reading off of Fig 3, the full model of macaque monkey cortex has a fraction of feedforward excitatory current in E cells of 10+%. Let us consider mouse V1 cortex about which much is known. The feedforward fraction of excitatory current has been estimated in mouse V1 to be approximately 30% [36]. This would place it near the 1/4 scaled down cortex in our catalogue. We can then make predictions about what mouse V1 cortex might be like based on our study of the 1/4 scaled-down model. One major prediction is that there should be few complex cells in mouse V1, from the results in Fig 5A. This prediction agrees with data [37]. Another prediction is different distributions of firing rates of E and I cells in mouse V1 from what is observed in macaque V1. Similar reasoning can be applied to other species.

#### Compensation mechanisms

Scaling down the number of neurons in the full cortical model caused the model to malfunction (Fig 1), and the downsized cortical models were brought into conformity with the full model’s firing rates and OS by the compensation algorithm we used (Table 1). The compensation algorithm worked largely by adjusting synaptic coupling weights to achieve criterion values of diff_*E*_, the difference between E and I synaptic currents that control E-cell firing, and diff_*I*_, the difference between E and I synaptic currents that control I-cell firing. The quantities diff_*E*_ and diff_*I*_ have traditionally been regarded as “small” or “negligible” in balanced state ideas [38]. Using them to steer firing rates into target ranges is, to our knowledge, novel.

Another question about this algorithm is, could a procedure along similar lines be employed by the real cortex to compensate for conditions that eliminated a large fraction of cortical neurons? We propose that synaptic plasticity as well as homeostatic synaptic scaling are known biological mechanisms that could implement the compensation algorithm we used. The scaling up of synaptic weights in neurons that are not firing enough has been observed in cortical cells in culture and in vivo in studies of homeostatic synaptic scaling [24], and the amounts of synaptic scaling demanded by the compensation algorithm from *n* ≤ 16 are comparable to the synaptic scaling that has been observed in experiments [24].

There are other known compensation mechanisms, e.g. Hebbian plasticity; see e.g. [39]. Our study was not intended as a study of compensation mechanisms for cortical tissues damaged through cell death or dysfunction, but similarities between such mechanisms and those used for bringing firing rates into range in scaled down cortical networks seem plausible, and studies of one will hopefully shed light on the other.

## Materials and methods

### Model equations and parameters

Consider a model neuron *n* of type *σ* = E or I in Layer 4*Cα*. The time evolution of its membrane potential is described by the leaky integrate-and-fire (LIF) equation

$$\begin{array}{c} \frac{dv}{dt}=-g_{R,\sigma }v-g_{E}\left(t\right)\left(v-V_{E}\right)-g_{I}\left(t\right)\left(v-V_{I}\right)\;. \end{array}$$ (2)

Here *v* is in normalized voltage units, the resting potential *V*_rest_ = 0 and spiking threshold *V*_th_ = 1. The membrane potential *v* is driven towards *V*_th_ = 1 by the LIF equation. When it reaches *V*_th_, a spike is fired, and *v* is reset to 0, where it is held for a refractory period of 2 ms before its dynamics resume. In the LIF equation, time *t* is in sec, the leakage conductance *g*_*R*,*σ*_ is set to 50 for *σ* = E and to 1.33*50 for *σ* = I (in units sec^−1^) [40]. The functions *g*_*E*_(*t*) and *g*_*I*_(*t*) are the E and I conductances of neuron *n* at time *t*; their time evolutions are described below. The constants *V*_*E*_ and *V*_*I*_ are the E and I reversal potentials, which in normalized coordinates are 14/3 and −2/3 respectively. The biophysical constants above are textbook [41].

The evolution of *g*_*I*_(*t*), the I conductance of neuron *n*, is given by

$$\begin{array}{c} g_{l}\left(t\right)\;=\;S^{\sigma I}\sum_{i\in P_{4C,I(n)}}\;\sum_{k=1}^{\infty }\;G_{\text{GABA}}\left(t-t_{k}^{i}\right)\;, \end{array}$$

where *S*^*σI*^ is the synaptic weight, or the constant describing the change in I-conductance in neuron *n* upon receiving synaptic input from an inhibitory neuron. Synaptic weights vary by ∼ 10% from neuron to neuron; for simplicity we write only their mean, which we assume depends only on the type *σ* of neuron *n*, hence the notation *S*^*σI*^. The first summation in the equation for *g*_*I*_(*t*) is over *P*_4*C*,*I*(*n*)_, the set of all I-neurons in layer 4*Cα* that synapse on neuron *n*. The second summation is over the spikes fired by neuron *i*. Specifically, $t_{k}^{i}$ is the time of the *k*th spike of neuron *i*. The function *G*_GABA_ (*s*) describes the time course of I-conductance for a neuron when a spike is fired by a presynaptic I-neuron at time *s* = 0. It is essentially an *α*-function with decay time ∼ 5 ms.

The E conductance *g*_*E*_(*t*) is the sum of 4 synaptic conductances coming from (I) LGN; (II) layer 4*Cα*; (III) layer 6; and (IV) “ambient”, a term into which we lump together the influence of multiple modulatory substances. We discuss each of these terms separately.

First,

$$\begin{array}{c} \left(I\right)\;:=\;S^{\sigma ,LGN}\sum_{i\in P_{LGN(n)}}\;\sum_{k=1}^{\infty }\;G_{\text{AMPA}}\left(t-t_{k}^{i}\right)\;. \end{array}$$

As above, *S*^*σ*,*LGN*^ is the synaptic weight from an LGN spike for a postsynaptic cell of type *σ*, *P*_*LGN*(*n*)_ is the set of LGN cells presynaptic to neuron *n*, and *G*_AMPA_ (*s*) is the conductance time course for AMPA. The decay time for AMPA is ∼3 ms.

Next,

$$\begin{array}{c} \left(II\right):=S^{\sigma E}\sum_{i\in P_{4C,E(n)}}\;\sum_{k=1}^{\infty }\left\{\rho_{A}^{\sigma }G_{\text{AMPA}}\left(t-t_{k}^{i}\right)+\rho_{N}^{\sigma }G_{\text{NMDA}}\left(t-t_{k}^{i}\right)\right\}\;. \end{array}$$

As before, *P*_4*C*,*E*(*n*)_ denotes the set of E-neurons in layer 4*Cα* that synapse on neuron *n*. Here $\rho_{A}^{\sigma }$ and $\rho_{N}^{\sigma }$ denote the fractions of synaptic input received by AMPA and NMDA receptors in a postsynaptic neuron of type *σ*; we used $\rho_{A}^{E}=0.8$, $\rho_{N}^{E}=0.2$, $\rho_{A}^{I}=0.67$, $\rho_{N}^{I}=0.33$. The decay time for *G*_NMDA_ is ∼ 80 ms. The feedback term (III) is identical to (II), except that *P*_4*C*,*E*(*n*)_ is replaced by *P*_6,*E*(*n*)_, the set of E-neurons in layer 6 that synapse on neuron *n*, and *S*^*σE*^ is replaced by $S_{6}^{\sigma E}$, a different number.

Finally, the term (IV) is modeled roughly (in the absence of more precise information) as

$$\begin{array}{c} \left(IV\right):=S^{amb}\sum_{Poisson,r_{\sigma }}G_{\text{AMPA}}\left(t-t_{k}\right)\;. \end{array}$$

Here “amb” is shorthand for “ambient”. The summation is over Poisson pulses, of size *S*^*amb*^ and at rate *r*_*σ*_, occurring at times *t*_*k*_. The times of occurrence of ambient pulses are independent from neuron to neuron.

With regard to layer 6, spontaneous spiking of L6 neurons was modeled as Poissonian, at 0.5–10 spikes/s [35]. When driven, if *f*_6,*max*_ represents the mean firing rate of a L6 neuron driven by its optimally oriented grating pattern at full contrast, we scaled this number down to about 1/4*f_6,*max*_ for the response to an orthogonal grating.

Finally, following [42], we assumed that each E-to-E spike from layer 4*Cα* carries a synaptic failure rate of up to 20%, and each Layer 6 spike carries a failure rate up to 50%.

#### Summary of parameter values used in the simulations in this paper

Coupling weight LGN → E-cells, *S*^*E*,*LGN*^ = *S*^EE^*2.425 = 0.055775.

Coupling weight LGN → I-cells, *S*^*I*,*LGN*^ = *S*^EE^*3.2 = 0.0736.

Coupling weight cortical E → E, *S*^*EE*^ = 0.023.

Coupling weight cortical E → I, *S*^*IE*^ = *S*^EE^*0.34 = 0.00782.

Coupling weight cortical I → E, *S*^*EI*^ = 0.046.

Coupling weight cortical I → I, *S*^*II*^ = 0.75*2*S^*EE*^ = 0.0345.

Coupling weight layer 6 → E, $S_{6}^{EE}=\left[0.00767,0.01533\right]$

Coupling weight layer 6 → I, $S_{6}^{IE}=0.003128$

Coupling weight ambient, *S*^*amb*^ = 0.01.

Ambient firing rate, $r_{E}^{A}=r_{I}^{A}=350$ Hz.

Peak driven firing rate in Layer 6, *f*_6,*max*_ ∼ 41.4 Hz.

### Algorithm for synaptic weight compensation

Below are the three steps of the algorithm for synaptic weight adjustment for the 1/*n*-cortex. All notations are as in the main text. With *n* fixed, we will omit the “*n*” in *m*_*Q*′*Q*_(*n*), writing only *m*_*Q*′*Q*_.

Step 1 *Seeking to produce desired values of E*_opt_ *and I*_opt_ *by adjusting S*^*Q*′*Q*^, *Q*, *Q*′ = *E*, *I, with the aim of producing for the* 1/*n-cortex diff*_*E*_ = 60 *and diff*_*I*_ = 120 *as in the full-size cortex*. Our scheme is to *assume* (i) *E*_opt_ and *I*_opt_ are the same as in the full-size cortex, and (ii) currents are proportional to number of spikes (neglecting the voltage dependence). We then compute *m*_*EE*_, *m*_*EI*_, *m*_*IE*_, and perform numerical simulations to locate a suitable *m*_*II*_.
  We compute *m*_*EE*_ and *m*_*EI*_ by solving
  $$\begin{array}{c} {\text{diff}}_{E}=60\;\text{and}\;\min\left(m_{EE},m_{EI}\right)=1\;. \end{array}$$
  In more detail, if *x* is the sum of the LGN and ambient currents, and *y*, *z* are the total E- and I-currents into an E-neuron in the optimally driven patch in the full-size cortex, then by assumption (ii) above, *m*_*EE*_ and *m*_*EI*_ are related by
  $$\begin{array}{c} x+\frac{1}{n}y\cdot m_{EE}-\frac{1}{n}z\cdot m_{EI}=60.\; \end{array}$$ (3)
  This is because in our scheme LGN and ambient inputs are unchanged. Total E-current produced within cortex is changed by the factor $\frac{1}{n}\cdot m_{EE}$ because there are 1/*n* as many E-neurons, which are assumed to fire at the same rate as before, and *S*^*EE*^ is altered by the factor *m*_*EE*_. Total I-current is changed similarly. This formula together with min(*m*_*EE*_, *m*_*EI*_) = 1 uniquely determines *m*_*EE*_ and *m*_*EI*_. (The “min” condition is arbitrary; there is a free parameter and we use it to keep *m*_*Q*′*Q*_ close to 1).
  Next we seek to achieve diff_*I*_ = 120. Here we use a formula analogous to (3) to calculate *m*_*IE*_ *assuming* *m*_*II*_ = 1. Then fixing the values of *m*_*EE*_, *m*_*EI*_, *m*_*IE*_ obtained, we search numerically around *m*_*II*_ = 1 for a suitable value of *m*_*II*_ to satisfy the firing rate requirements (a)–(c).
  We pause to explain why Step 1 may fail to produce *E*_opt_ and *I*_opt_ in the desired range (for any value of *m*_*II*_). This is because we have operated under a number of assumptions that are only approximately true, one of which being that *E*_opt_ is determined by diff_*E*_ and *I*_opt_ by diff_*I*_. From the LIF Eq (2), we see that this assumption is true modulo the leak term (the first term in the equation). Because of the leak, how much of the current is converted into spikes and how much of it is ineffectual depends on input patterns as well as firing rates. For example, 50 spikes evenly spaced at 20 ms apart are much less effective in producing spikes than 50 spikes arriving in relatively quick succession, as the latter will drive the membrane potential over threshold multiple times before the synaptic current leaks away.
  Thus to produce the same *E*_opt_ and *I*_opt_, the values of diff_*E*_ and diff_*I*_ needed in the 1/*n*-cortex are not necessarily the same as those in the full-size cortex, and diff_*E*_ = 60 and diff_*I*_ = 120 were just approximate values to start from.
Step 2 *Successive adjustments of diff_*E*_ and diff_*I*_*. When Step 1 fails to produce *E*_opt_ and *I*_opt_ in the desired range, we perform the following adjustment using the information gleaned from Step 1: If *E*_opt_ is too high, either reduce diff_*E*_ or increase diff_*I*_; and if it is too low, either increase diff_*E*_ or reduce diff_*I*_. Do the same for *I*_opt_. Then repeat Step 1, and hope that successive adjustments will bring *E*_opt_ and *I*_opt_ into the desired range.
Step 3 *Increasing OS by exchanging S*^*IE*^ *for S*^*I*,*LGN*^. To increase OS, we replaced cortical excitation of inhibitory neurons, which is reduced in scaled-down cortices, with increased LGN excitation. This was done by exchanging *S*^*IE*^ for *S*^*I*,*LGN*^ so that the net E-current term in diff_*I*_ for neurons in the optimal patch remained unchanged. The idea is that the portion of E-drive from cortex into I-neurons is larger in the optimal than in the orthogonal patch because of higher E-neuron firing rate in the optimal patch, so the increase in *S*^*I*,*LGN*^ that perfectly balances the decrease in *S*^*IE*^ in the optimal patch will result in a net increase of total E-input to I-neurons in the ortho patch. This produces stronger I-firing in the ortho patch; that reduces *E*_ortho_, thereby increasing OS.
  While increasing LGN excitation of I cells is an effective tool for increasing OS, we point out that the amount of *S*^*IE*^ and *S*^*I*,*LGN*^ exchanged cannot be arbitrarily large, as it increases the ratio *I*_bg_/*E*_bg_ at the same time, so that increasing OS this way can cause spontaneous activity to drop below acceptable levels.

**Fig 7:**
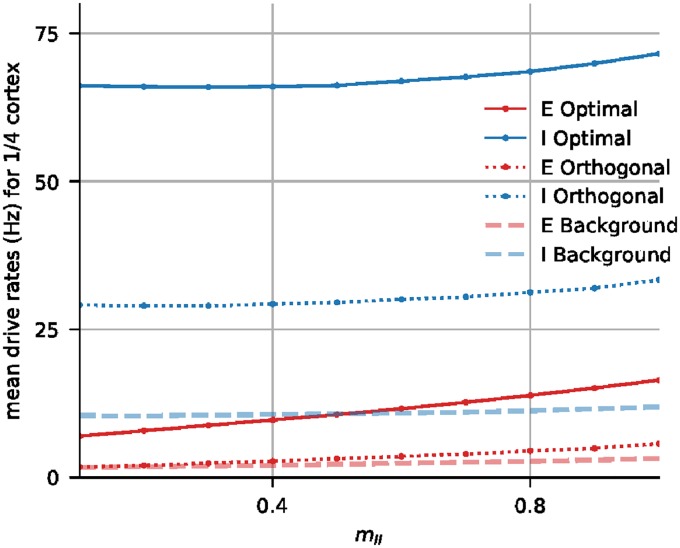
Plots of *E*_opt_, *I*_opt_, *E*_ortho_, *I*_ortho_, *E*_bg_ and *I*_bg_ as functions of *m*_*II*_ for the 1/4-cortex, after *S*^*I*,*LGN*^ was increased by 10%. From the graphs plotted, we determined that *m*_*II*_ = 0.8 was acceptable with regard to the conditions stipulated in **Results (Restoring firing rates through synaptic compensation)**.

This completes the description of the algorithm we used. An example of the simulations performed in search of a suitable *m*_*II*_ is shown in Fig 7.

We remark that for the smaller model cortices, extra care was needed to ensure that connectivities within local populations were not unduly biased. Connectivities in our models were decided by coin tosses together with modification to narrow the range to essentially 1 standard deviation as in [9]. For the larger cortices the law of large numbers ensured that distributions of numbers of presynaptic neurons were reasonable. This is not always the case with the smaller cortices. In the 1/49-model cortex, for example, optimal patches such as those we used to compute *E*_opt_ and *I*_opt_ contained only ∼ 10 neurons, for which the mean number of presynaptic I-neurons was 2. When dealing with such small numbers, variances can be large: In the example above, “unlucky draws” in which a few neurons picked only one or zero presynaptic I-neurons can have a dramatic effect on model performance. When that happened, we redrew until deviation from the mean became acceptable.

All simulations were performed using the Python library Brian2 [43], using time-steps of 0.01 ms.

## Supporting information

S1 Dataset Fig 1 data. Contains E cell data for Fig 1, left panel.
(CSV)

S2 Dataset Fig 1 data. Contains I cell data for Fig 1, left panel.
(CSV)

S3 Dataset Fig 1 data. Contains data for Fig 1, right panel.
(CSV)

S4 Dataset Fig 2 data. Contains data for row 0, column 0 of Fig 2.
(CSV)

S5 Dataset Fig 2 data. Contains data for row 0, column 1 of Fig 2.
(CSV)

S6 Dataset Fig 2 data. Contains data for row 0, column 2 of Fig 2.
(CSV)

S7 Dataset Fig 2 data. Contains data for row 0, column 3 of Fig 2.
(CSV)

S8 Dataset Fig 2 data. Contains data for row 0, column 4 of Fig 2.
(CSV)

S9 Dataset Fig 2 data. Contains data for row 1, column 0 of Fig 2.
(CSV)

S10 Dataset Fig 2 data. Contains data for row 1, column 1 of Fig 2.
(CSV)

S11 Dataset Fig 2 data. Contains data for row 1, column 2 of Fig 2.
(CSV)

S12 Dataset Fig 2 data. Contains data for row 1, column 3 of Fig 2.
(CSV)

S13 Dataset Fig 2 data. Contains data for row 1, column 4 of Fig 2.
(CSV)

S14 Dataset Fig 2 data. Contains data for row 2, column 0 of Fig 2.
(CSV)

S15 Dataset Fig 2 data. Contains data for row 2, column 1 of Fig 2.
(CSV)

S16 Dataset Fig 2 data. Contains data for row 2, column 2 of Fig 2.
(CSV)

S17 Dataset Fig 2 data. Contains data for row 2, column 3 of Fig 2.
(CSV)

S18 Dataset Fig 2 data. Contains data for row 2, column 4 of Fig 2.
(CSV)

S19 Dataset Fig 2 data. Contains data for row 3, column 0 of Fig 2.
(CSV)

S20 Dataset Fig 2 data. Contains data for row 3, column 1 of Fig 2.
(CSV)

S21 Dataset Fig 2 data. Contains data for row 3, column 2 of Fig 2.
(CSV)

S22 Dataset Fig 2 data. Contains data for row 3, column 3 of Fig 2.
(CSV)

S23 Dataset Fig 2 data. Contains data for row 3, column 4 of Fig 2.
(CSV)

S24 Dataset Fig 3 data. Contains data for Fig 3.
(CSV)

S25 Dataset Fig 4 data. Contains data for Fig 4.
(CSV)

S26 Dataset Fig 5 data. Contains data for CV plots in Fig 5.
(CSV)

S27 Dataset Fig 5 data. Contains data for E-neuron rasters in row 0 of Fig 5.
(CSV)

S28 Dataset Fig 5 data. Contains data for E-neuron rasters in row 1 of Fig 5.
(CSV)

S29 Dataset Fig 5 data. Contains data for E-neuron rasters in row 2 of Fig 5.
(CSV)

S30 Dataset Fig 5 data. Contains data for I-neuron rasters in row 0 of Fig 5.
(CSV)

S31 Dataset Fig 5 data. Contains data for I-neuron rasters in row 1 of Fig 5.
(CSV)

S32 Dataset Fig 5 data. Contains data for I-neuron rasters in row 2 of Fig 5.
(CSV)

S33 Dataset Fig 5 data. Contains E cell data for Fig 5.
(CSV)

S34 Dataset Fig 5 data. Contains I cell data for Fig 5.
(CSV)

S35 Dataset Fig 6 data. Contains data for Fig 6.
(CSV)

S36 Dataset Fig 7 data. Contains data for Fig 7.
(CSV)

S1 Text Dataset documentation. Documentation for datasets in Supporting Information.
(DOCX)
