# Supplementary material for: A case study in the functional consequences of scaling the sizes of realistic cortical models
Source: PLoS Comput Biol. 2019 Jul 23;15(7):e1007198. doi: 10.1371/journal.pcbi.1007198 (PMC6677387; doi:10.1371/journal.pcbi.1007198)
Supplement: S1 Text — Documentation for datasets in Supporting Information. (DOCX) [file pcbi.1007198.s037.docx]

The following is a description of the data files, which correspond to the figures of the paper.

**Fig 1 datasets**

Left panel: For datasets S1 and S2, the first column denotes the size of the cortex. The subsequent columns denote the Eopt and Iopt values, respectively, using various random seeds.

Right panel: for S3_dataset.csv, the first column denotes the size of the cortex. The subsequent columns (as named) denote the Eopt and Eortho values.

For plotting the figures, the x axis is set to a log scale.

**Fig 2 datasets**

Datasets S4-23 contain peak firing rate data for rows 0-3, columns 0-4 of Fig 2. S4 corresponds to row 0, column 0, and subsequent datasets proceed S5: row 0, column 1; S6: row 0, column 2; S7: row 0, column 3; S8: row 0, column 4; S9: row 1, column 0; etc. For each file, a given matrix entry denotes the peak firing rate corresponding to a specific orientation (denoted by the first column) and a specific spatial frequency (denoted by the subsequent columns).

**Fig 3 datasets**

For S24, the rows denote various current sources (0: ambient, 1: LGN, 2: Layer 6, 3: Layer 4Calpha) and the columns denote the various model cortices, as indicated in the column names.

**Fig 4 datasets**

Dataset S25 corresponds to Fig 4. The first column denotes time in ms, and the subsequent columns denote voltage, E current, I current, for the full, 1/4 and 1/16 cortices, as indicated in the column name.

**Fig 5 datasets**

Dataset S26 provides the CV distributions, with the first column being the bin centers and subsequent columns denoting the corresponding bin values for the full, 1/4, 1/16 cortices as indicated in the column name.

Datasets S27-29 provide E neuron raster data for rows 0, 1, 2. For each raster file, the 2 columns denote the neuron spike index and spike time as indicated by the column names. The spike time ranges from 3000 to 3500 ms.

Datasets S30-32 provide I neuron raster data for rows 0, 1, 2 (formatting is the same as in S27-29).

Datasets S33 and S34 contain the mean E and I firing rate distributions, respectively. For each of the files, the first column denotes the bin centers and the subsequent columns denote the corresponding bin values for the full, 1/4, 1/16 cortices as indicated in the column name.

The number of silent E, I neurons for the full, 1/4, 1/16 cortices is,

Full cortex: silent E = 11, silent I = 3

1/4 cortex: silent E = 14, silent I = 0

1/16 cortex: silent E = 9, silent I = 3

**Fig 6 datasets**

For Fig 6 (dataset S35), each of the histograms has 16 bins. The first column corresponds to the preferred grating, the rest of the columns are the values in the bins for each of the histograms (with the columns named accordingly).

**Fig 7 datasets**

For Fig 7 (dataset S36), the first column corresponds to the mII values, the other columns to the y values for the various curves (with these columns named based on the legend labels).
